# Calculation Based on the Formation of Mg_2_Si and Its Effect on the Microstructure and Properties of Al–Si Alloys

**DOI:** 10.3390/ma14216537

**Published:** 2021-10-30

**Authors:** Jianfei Hao, Baoyi Yu, Jiancong Bian, Bin Chen, Huishu Wu, Weirong Li, Yanfang Li, Runxia Li

**Affiliations:** 1 School of Material Science and Engineering, Shenyang University of Technology, Shenyang 110870, China; gdhaojianfei@163.com (J.H.); baoyiy@163.com (B.Y.); bianjc1993@163.com (J.B.); 2 School of Material Science and Engineering, Dongguan University of Technology, Dongguan 523808, China; chenbin@dgut.edu.cn (B.C.); ggwuhuishu@163.com (H.W.); 3 Dongguan Eontec Co., Ltd., Dongguan 523662, China; liwr@e-ande.com (W.L.); PUR@meion.com.cn (Y.L.)

**Keywords:** phase diagram calculation, Mg_2_Si phase, microstructure evolution, mechanical properties, Al−17Si−4Cu−(0.5, 2.5)Mg alloy

## Abstract

In order to investigate the effect of Mg_2_Si formation on the microstructure and properties of an Al−Si alloy, the critical point of a hypereutectic Al−17Si−4Cu−Mg alloy was calculated by Pandat software. The calculation results of the equilibrium phase diagram show that the critical point for Mg_2_Si phase formation for the alloy was obtained when the Mg content was 2.2%. The contents of 0.5 wt.% Mg and 2.5 wt.% Mg were selected as the research object. The content of Mg increased from 0.5 wt.% to 2.5 wt.%, the eutectic Si in the matrix was reduced, and the Chinese character-like Mg_2_Si phase appeared in the microstructure. In the peak ageing state, in addition to θ″ and Q′ phases that were mainly precipitated, there was also needle-like β″ precipitation in the 2.5 wt.% Mg content alloy. Larger precipitates were found in 2.5 wt.% content alloys, mainly due to the promotion of the solid solution having the aggregation and segregation of more solute elements in the matrix. The tensile strength, elongation, and hardness of hypereutectic Al−17Si−4Cu−0.5Mg alloy under peak ageing were 331 MPa, 3.11%, and 152.1 HB, respectively. The tensile strength and the elongation decreased while the hardness increased with the 2.5 wt.% Mg content, which is due to the formation of hard and brittle Mg_2_Si and Al_8_FeMg_3_Si, which has a splitting effect on the matrix.

## 1. Introduction

Hypereutectic Al–Si alloys have been extensively utilized in the automotive industry for manufacturing large and complex castings, such as engine cylinder heads, engine blocks, or gearbox housings, because of the desirable integration of their favorable casting, cost-effectiveness, and corrosion resistance [1,2,3,4]. Si is almost insoluble in the matrix; only eutectic or primary Si can be formed in the matrix. Si phases are highly brittle, which causes a decrease in the strength of aluminum alloys. Thus, Al−Si alloys have less desirable mechanical performances. For enhancing Al−Si alloys’ mechanical performances, an appropriate amount of alloy elements will usually be added to the alloy and a reasonable heat treatment process will be adopted. Cu is the most common alloy element added in an Al−Si alloy, Cu is added to form θ-Al_2_Cu and other phases in the alloy and then, through ageing and strengthening of these phases, alloy properties are improved [5,6].

In addition to Cu, the addition of Mg has a great influence on the microstructure and properties of Al−Si alloys, Al_5_Cu_2_Mg_8_Si_6_, Mg_2_Si, and some other second phases are usually formed in Al−Si−Cu−Mg quaternary alloys in an as-cast state. The Mg_2_Si phase has a low density (1.90 g/cm^3^), a high melting point (1087 °C), a high hardness (4.5 × 10^9^ N/m^2^), a high elastic modulus (120 GPa), and a low thermal expansion coefficient (7.5 × 10^−6^ K^−1^) [7]. Therefore, the formation of Mg_2_Si is very important to the mechanical properties of hypereutectic Al−Si−Cu−Mg alloys. A.R. Farkoosh et al. [8] studied that the strength of Al−Si−Cu alloys have been significantly improved after adding the Mg element (in the range of 0.3–0.5 wt.%) at 300 °C, the 0.2% proof stress increased from 35 MPa to 58.5 MPa. While K. S. Alhawari et al. [9] found that the plasticity of Al−6Si−3Cu alloy decreased significantly after adding an appropriate amount of Mg (in the range of 0.3–2 wt.%), the elongation decreased from 3.2% to 0.6%. Yang et al. [10] found that Mg content can be controlled at a level up to 0.73 wt.% by increasing the strength with acceptable ductility under as-cast and heat treatment conditions in the Al−Si−Cu alloy. Some studies also showed that the addition of Mg will affect the microstructure of an Al alloy. M.A.M. Arif et al. [11] found that after adding 1.2 wt.% Mg to 2014 aluminum alloy, an increase in Mg content used also resulted in the formation of the compact π−Al_8_FeMg_3_Si_6_ phase and a decrease in the amount of the sharp and plate-like structures of the β-Al_5_FeSi phase. The addition of Mg mainly affects the formation of Mg_2_Si and the distribution of solute elements in the matrix. Ni Tian et al. [12] found that the volume of Mg_2_Si was gradually increased, while the divorced eutectic phenomenon of the quaternary eutectic structure was weakened with the increase in Mg content. After T6 treatment, the fine dispersed β(Mg_2_Si) phase and the Q(Al_5_Cu_2_Mg_8_Si_6_) phase precipitated successively, which played a dispersion strengthening role in the alloy. The precipitation sequence and precipitation type of the second phase are closely related to the addition of alloy components, and the size and distribution of precipitates are closely related to the subsequent heat treatment process and forming process [13,14]. Our research group has studied that the mechanical properties of the alloy will be significantly improved after semi-solid extrusion [15]. Thus, in order to further expand the application of Mg_2_Si formation in a cast Al−Si alloy, it is very valuable to study the distribution of Mg_2_Si in an Al−Si−Cu−Mg alloy and the effect of Mg_2_Si formation on the mechanical properties of the alloy under semi-solid extrusion process.

Many scholars have studied the influence of Mg content on the microstructure and mechanical properties of hypoeutectic Al−Si alloys [7,8,9,10,11]. However, the quantitative calculation of the equilibrium phase diagram formed by the Mg_2_Si phase in the hypereutectic Al−Si−Cu−Mg alloy and the linear relationship between the Mg element and the Cu element are fewer. With the development of phase diagram calculation software and the improvement of databases, phase diagram calculation software is widely used to calculate the equilibrium phase diagram and solidification process of target alloys and conduct preliminary screening based on it, so as to narrow the selection range and improve the efficiency [16,17]. At present, many phase diagram calculation software have appeared in the field of material science, such as Thermo-cale, Fact, Pandat, Luka and Mtdata. The biggest advantage of Pandat is that it is easy to operate. It can automatically search for the stable phase of the multi-phase system without pre-setting the initial value and supports user−defined databases. Meanwhile, the system optimizes the local minimum in the free energy minimum algorithm. It provides a powerful calculation platform for phase diagram and thermodynamic calculation.

In this paper, the equilibrium point of Mg_2_Si phase formation in hypereutectic Al−Si−Cu−Mg alloy are first calculated by the equilibrium phase diagram, and the effects of the formation of Mg_2_Si on the microstructure, solidification path, ageing precipitate behavior and mechanical properties of the alloy were analyzed. To explore the changes in the number and morphology of the precipitated phases of the alloy, we conducted transmission electron microscopy (TEM) on our prepared Al−Si−Cu−xMg alloys.

## 2. Experiment

### 2.1. Phase Diagram Calculation

The equilibrium phase diagrams of an Al−17Si−4Cu−xMg alloy with two different amounts Mg content were calculated by Pandat software. Al−data was selected for the data, and the pressure was set to 101.325 KPa. Mg was added to Al−17Si−4Cu alloys to fabricate two hypereutectic Al−17Si−4Cu−xMg alloys (x = 0.5 and 2.5, mass fraction, %). Al−Si−Cu−Mg alloys were synthesized using commercial ingots containing pure Mg/Al, Al−50 wt.% Cu, and Al−30 wt.% Si. Table 1 presents alloy chemical compositions.

### 2.2. Material Manufacture

In the experimental processes, the weights of all elements were measured to specific proportions using diverse additional contents to compensate for burning loss in the process of melting. The alloys were melted within the silicon carbide crucible placed within the 750 ± 5 °C electrically resistant furnace. Pure Mg at a certain content was preheated to 300 °C at an argon atmosphere. Before extrusion, a small amount of the melt was taken for composition analysis. X-ray fluorescence (XRF) was conducted to determine alloy chemical compositions.

When the alloy compositions were analyzed and the sample was skimmed, the semisolid slurry was prepared, and extrusion was conducted. We maintained the melted liquid for 20 min under 670 °C, which was later poured into the crucible. The semisolid slurry was prepared using ultrasonic vibration technology. The vibration time was 40 s, and k-type thermocouple was utilized to measure the temperature at which the slurry was poured, which was 600 °C. Subsequently, the 4500KN HPDC machine was employed to load the melt into the shot sleeve for casting.

The alloy was subjected to ageing and solid solution treatments at an extruded state. Relevant phase diagrams and references were used to determine the heat treatment of the alloys [18,19]. In this experiment, the solution time and temperature were 525 °C and 10 h, and the ageing time and temperature were 175 °C and 0–24 h, respectively.

### 2.3. Material Characterisation

Scanning electron microscopy (SEM) and optical microscopy (OM) were employed for characterizing the as-cast alloys to detect microstructural changes. The JEMTM 2000EX Ⅱ transmission electron microscope was utilized for conventional TEM analysis by the use of an energy-dispersive X-ray spectroscopy (EDS) instrument. For preparing thin foils for TEM examination, sample slices were subjected to mechanical grinding and cutting to 3 mm discs. For the prepared alloys, we determined their phase compositions by X-ray diffractometry (XRD-7000). The solidification behavior was analyzed through differential scanning calorimetry (DSC). The JXA-1720 electron probe X-ray microanalyzer (EPMA) was utilized to measure solute element distribution.

We evaluated the T6-treated and as-cast alloys for their mechanical performances through tensile and hardness tests. The tensile speed of the sample was 0.1 mm/s, the size of the Brinell hardness sample was 12 mm × 3 mm, pressure holding time was 30 s, the diameter of the indenter was Φ 2.5 mm, and the load was 62.5 kg. Results were the averages obtained from 6 measurements.

## 3. Results and Discussion

### 3.1. Phase Diagram for the As-Cast Al−17Si−4Cu−xMg Alloys

**Figure 1:**
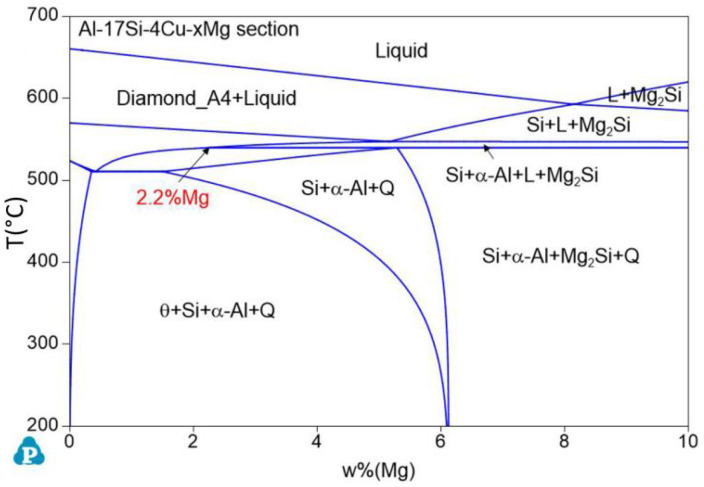
Equilibrium phase diagram for the Al−17Si−4Cu−xMg alloys calculated using Pandat software.

Figure 1 presents the equilibrium solidification phase diagram of the semisolid extrusion Al−17Si−4Cu−xMg alloys. This phase was obtained with Pandat software. For the hypereutectic Al−17Si−4Cu alloy, at an Mg level of 2.2 wt.%, the figure is divided into two parts. At an Mg level of <2.2 wt.%, the main second phase in the alloy is the α-Al, Si, θ-Al_2_Cu, Q-Al_5_Cu_2_Mg_8_Si_6_ quaternary phase. In addition, the Q-Al_5_Cu_2_Mg_8_Si_6_ quaternary phase is formed in the alloy structure before the θ-Al_2_Cu phase. At an Mg level of >2.2 wt.%, the Q-Al_5_Cu_2_Mg_8_Si_6_ phase directly solidifies from the liquid before the θ-Al_2_Cu eutectic is formed, and the solidified structure comprises a small Mg_2_Si phase.

**Figure 2:**
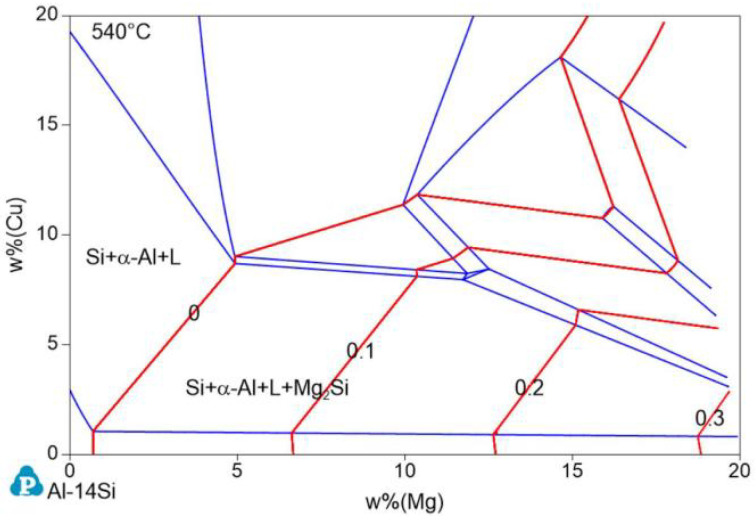
Phase diagram of the isothermal cross section of the Al−17Si−Cu−Mg alloy system at 540 °C.

The isothermal cross section of Al−17Si−Cu−Mg at 540 °C was calculated according to the minimum temperature for the stable existence of the Mg_2_Si phase, that is, 539.6 °C (Figure 2). In Figure 2, the red line is the Mg_2_Si content isoline, and the number marked on it represents the Mg_2_Si content. When the Cu and Mg contents are <10 and <5 wt.%, respectively, the coordinates of the two ends of the zero−content line of Mg_2_Si are (0.69 Mg and 1.02 Cu) and (4.90 Mg and 8.69 Cu), respectively. Therefore, the line equation is given as Formula (1):

(1) w% (Cu)=−0.24+1.82 w% (Mg)

When the actual Cu content ratio is less than the wt.% (Cu) value calculated with this equation, Mg_2_Si is formed. Substituting the measured Mg content of 2.45% in the experiment, Cu wt.% becomes 4.31%. The actual addition amount of Cu is 3.97%, which is lower than the critical value and, theoretically, the Mg_2_Si phase can be produced.

**Figure 3:**
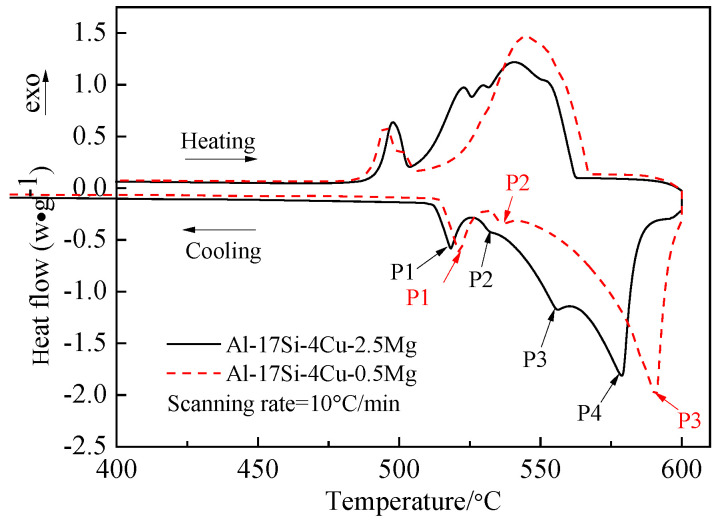
DSC heating curves for the semisolid Al−17Si−4Cu−xMg alloys.

**Figure 4:**
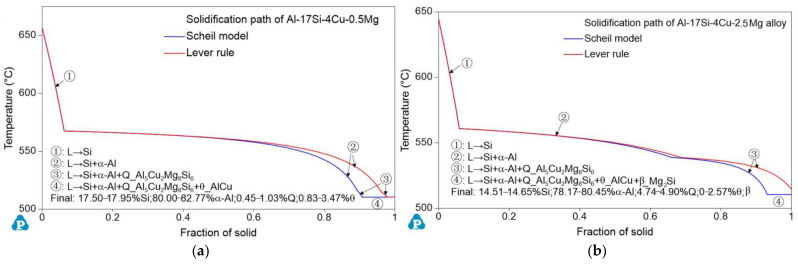
Solidification path of the Al–17Si–4Cu–xMg alloy with temperature in Al–17Si–4Cu alloy solidification containing (**a**) 0.5 Mg and (**b**) 2.5 Mg.

Figure 3 presents the DSC curves of the hypereutectic Al−17Si−4Cu−0.5Mg and Al−17Si−4Cu−2.5Mg alloys subjected to semisolid extrusion. The DSC analysis was conducted to identify the solidification behavior. Table 2 presents temperatures associated with all peaks present in the DSC curve. The DSC results showed that liquidus and eutectic temperatures decreased as Mg level increased. There were three peaks in the Al−17Si−4Cu−0.5Mg alloy and four peaks in the Al−17Si−4Cu−2.5Mg alloy, marked as P1, P2, P3, and P4. Figure 4 shows the solidification path of the two alloys. From the DSC curve and solidification path, the solidification behavior of the two alloys can be obtained. According to DSC solidification curve and solidification path diagram, the alloy solidification path can be seen as follows: first, α-Al is formed, followed by the Al−Si eutectic. When the temperature further decreases, Q phase is formed by the ternary eutectic reaction via the rest liquid reaching its composition; in this regard, Q phase is distributed surrounding the eutectic Si, and excessive Cu transforms into the Al_2_Cu phase. However, for the hypereutectic Al−Si−Cu−Mg alloy that contained a high Mg amount, in addition to Q-phase formation, the remaining Mg combines with Si. Mg_2_Si forms after the Al−Si eutectic reaction. Therefore, for the Al−17Si−4Cu−0.5Mg alloy, three peaks P1, P2, and P3 correspond to Al_2_Cu phase, Q phase, and eutectic Si phase and α-Al, respectively. For the Al−17Si−4Cu−2.5Mg alloy, the additional peak P4 corresponds to the Mg_2_Si phase. Solidification temperature decreases with an increase in the Mg content.

**Figure 5:**
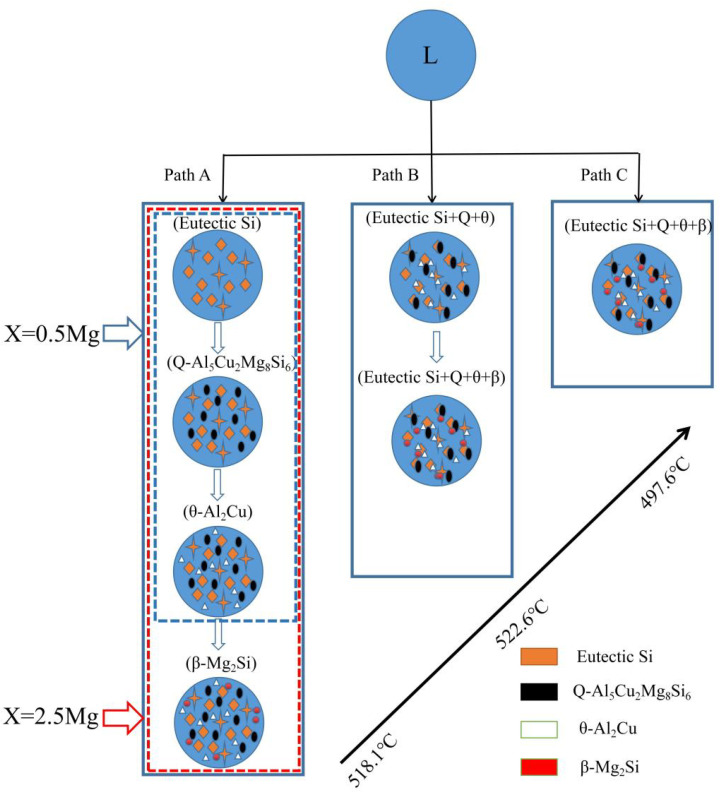
Solidification paths of Al−17Si−4Cu−xMg alloys.

Figure 5 illustrates the schematic solidification of the hypereutectic Al-17Si-4Cu-xMg alloy. From the DSC analysis and solidification path, the process of Al-17Si-4Cu-0.5Mg alloy solidification includes: L → L1 + α-Al → L1 + α-Al + eutectic Si → L1 + α-Al + eutectic Si + Q-Al_5_Cu_2_Mg_8_Si_6_ + θ-Al_2_Cu. The process of Al-17Si-4Cu-2.5Mg alloy solidification includes: L → L1 + α-Al → L1 + α-Al + eutectic Si → L1 + α-Al + eutectic Si + Q-Al_5_Cu_2_Mg_8_Si_6_+ θ-Al_2_Cu → L1 + α-Al + eutectic Si + Q-Al_5_Cu_2_Mg_8_Si_6_ + θ-Al_2_Cu + β-Mg_2_Si. Table 3 is the microstructure of the alloy after solidification corresponding to different Mg content calculated by the Scheil module.

### 3.2. Microstructure of the Al−17Si−4Cu−xMg Alloys

**Figure 6:**
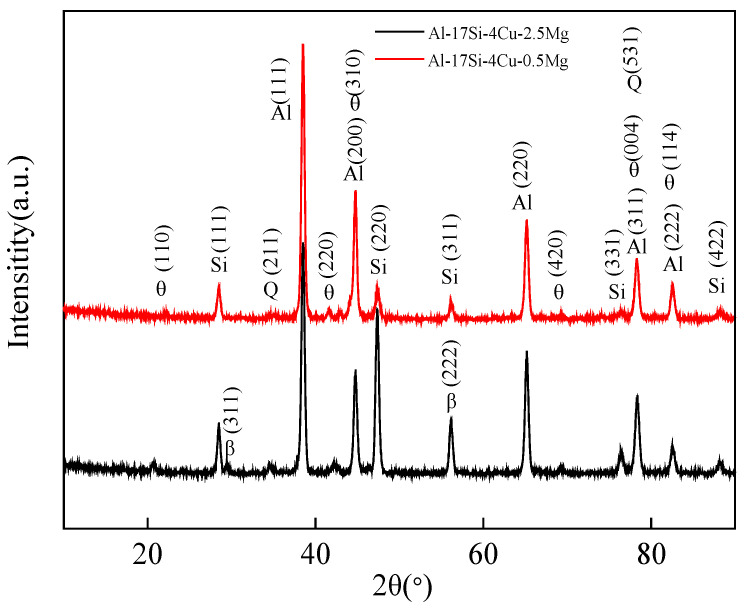
XRD curves for Al−17Si−4Cu−xMg alloys.

Figure 6 presents the XRD curves of the hypereutectic Al−17Si−4Cu−0.5Mg and Al−17Si−4Cu−2.5Mg alloys obtained during semisolid extrusion formation. The XRD curves reveal that the peak positions of the basic second phases in the two alloys are the same. The diffraction peak intensity of the Q phase shows an increasing trend, as with the Mg level. In an XRD curve, a hypereutectic Al−Si−Cu−Mg alloy with a higher Mg content shows one additional peak corresponding to the Mg_2_Si phase than that with a lower Mg content.

**Figure 7:**
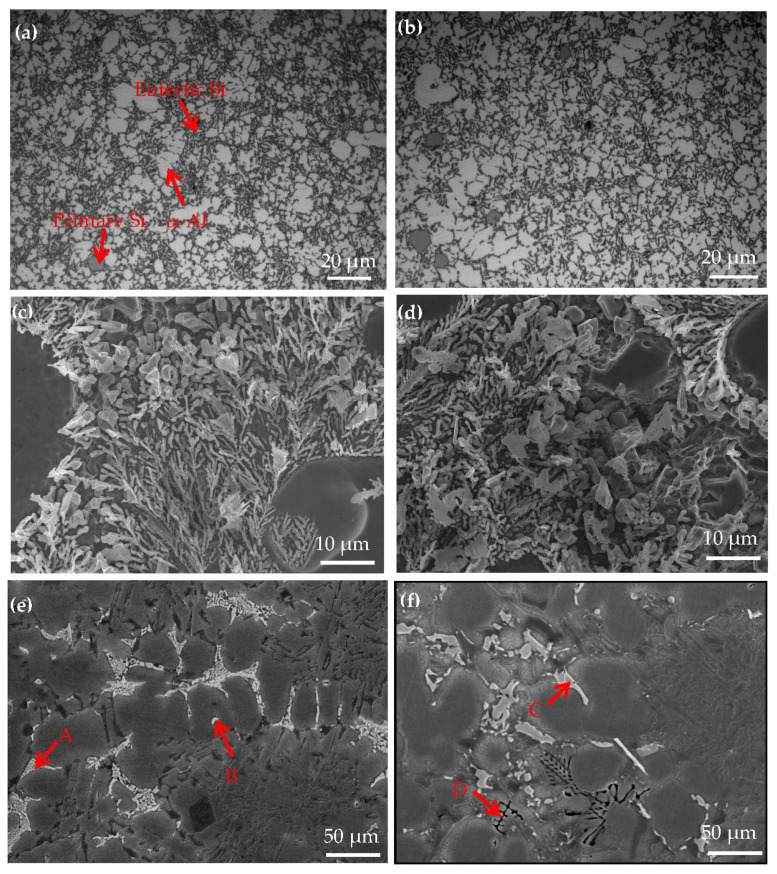
Microstructure of the Al−17Si−4Cu−xMg alloys. (**a**) OM microstructure, (**c**) High magnification SEM microstructure and (**e**) LOW magnification SEM microstructure of Al−17Si−4Cu−0.5Mg; (**b**) OM microstructure, (**d**) High magnification SEM microstructure and (**f**) LOW magnification SEM microstructure of Al−17Si−4Cu−2.5Mg.

Figure 7 shows the OM and SEM images for hypereutectic Al−17Si−4Cu−0.5Mg and hypereutectic Al−17Si−4Cu−2.5Mg alloys obtained during the semisolid extrusion formation. Table 4 presents EDS analyses on the second phase. The addition of Mg slightly affects the microstructure of the two alloys (Figure 7). In a high−Mg (2.5 wt.%) (Figure 7b) alloy microstructure, α-Al dendrites slightly increased relative to the low−Mg (0.5 wt.%) (Figure 7a) alloy microstructure. Meanwhile, compared with a low−Mg alloy (Figure 7c), the size of eutectic Si decreases, but the morphology is coarsened slightly in the high-Mg alloy (Figure 7d). Figure 7e,f illustrates the SEM structure of two alloys formed through semisolid extrusion. According to the EDS results, the low−Mg alloy mainly comprises a white, short, rod-like Al_2_Cu phase (marked as B) and an Al_5_Cu_2_Mg_8_Si_6_ quaternary phase (marked as A) (Figure 7e). For high−Mg alloys, apart from the second phase, both white acicular and black skeletal phases could be observed within the matrix. EDS analyses on two second phases showed that the white strip and black skeletal phases were the Al_8_FeMg_3_Si (marked as C) and Mg_2_Si (marked as D) phases, respectively (Figure 7f). This result conformed to calculations for the equilibrium solidification phase diagram and XRD curve analysis.

**Figure 8:**
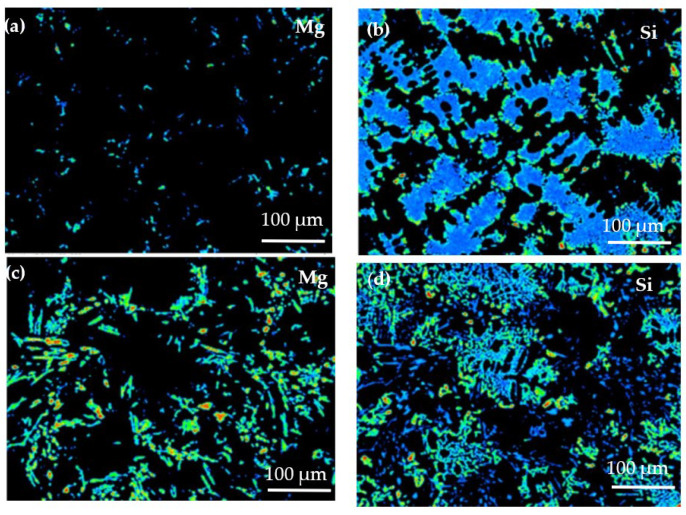
Distribution analysis of Mg and Si through EPMA: (**a**) and (**b**) Al−17Si−4Cu−0.5Mg, (**c**) and (**d**) Al−17Si−4Cu−2.5Mg.

Figure 8 shows the EPMA analysis results of the solute elemental distribution of the two alloys. Due to its low solubility in the matrix at room temperature, Mg is distributed on the matrix surface. In the Al−17Si−4Cu−0.5Mg alloy (Figure 8a), Mg is dispersed in the matrix in the form of dots, and the energy count is low. In the Al−17Si−4Cu−2.5Mg alloy (Figure 8c), Mg is distributed in the form of round dots and long rods. According to the distribution bar of energy counts present on the right side in Figure 8, the Mg energy count is large in some areas. Si available in the structure provides a sheet distribution with low Mg content (Figure 8b). However, for an alloy that contains 2.5 wt.% Mg, Si provides grid distribution (Figure 8d). This phenomenon occurs because an excessive amount of Mg consumes a part of the Si. Mg atoms accumulated during solidification tend to gather in the front of the eutectic Si phase solidification interface; the alloy has decreased eutectic temperature. The decrease in eutectic temperature can lead to a decrease in the Si phase growth rate, but it mainly causes the decreased solidification efficiency, which leads to coarsening of the eutectic Si phase.

**Figure 9:**
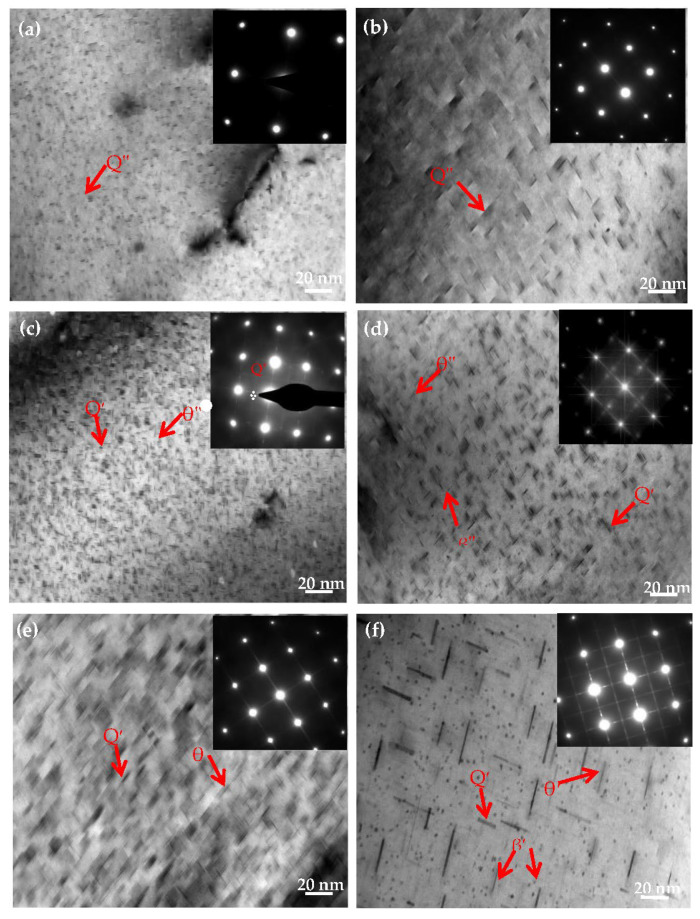
TEM images of the formed semisolid hypereutectic Al−17Si−4Cu−xMg alloys at different ageing times, and associated SADP of [001]Al: (**a**,**b**) under aged, (**c**,**d**) peak aged, (**e**,**f**) over aged.

To further explore the effects of Mg contents on the Al−Si−Cu alloy microstructure, the Al−17Si−4Cu−xMg alloy was analyzed through TEM in the direction of [001]Al after the T6 heat treatment. From the literature, the ageing time was selected as 2, 6, and 24 h [20,21]. Figure 9a,c,e illustrates the TEM microstructure of the Al−17Si−4Cu−0.5Mg alloy under a different ageing time; the insets present the corresponding selected area diffraction pattern (SADP). At an early ageing stage, a fine and dispersed point-like phase and short rod-like precipitates were observed in the matrix. The electron diffraction pattern obtained from the [001] direction presents the diffraction characteristics of the GP zone (Figure 9a) [22,23,24]. At the peak ageing stage, the number of precipitates increased, and numerous acicular precipitates, which were confirmed to be θ″ by using SADP, appeared in the microstructure (Figure 9c). In the over ageing stage, acicular precipitates continued to grow (Figure 9e); the precipitate length was 20–30 nm.

Figure 9b,d,f shows TEM structure for the Al−17Si−4Cu−2.5Mg alloy at different ageing times. The precipitate size in the alloy with higher Mg (2.5 wt.%) is larger than that in the alloy with lower Mg (0.5 wt.%). In peak ageing, numerous needle-like phases were observed in the crystal. According to the cross-star awn in the diffraction spots in Figure 9d, the precipitate exhibited the metastable strengthening phase β″ [25,26,27]. The β″ phase mainly precipitated along the [001]Al direction. The TEM image indicates that the β″ precipitates, in this direction, exhibit needle-like and spherical morphologies. In the over-ageing stage, thin plate θ′ precipitates were associated with planes {001} (Figure 9f). In the SADP pattern (inset off Figure 9f), its streaking down the 002 systematic rows conforms to thin precipitates. Alongside this, the precipitates with θ′-Al_2_Cu plate shape showed a representative orientation relation to the aluminum matrix (001)θ′∥(001)Al and [100]θ′∥ [100]Al [14,24].

Under high pressure, the solute diffusion coefficient D can be described as Formula (2) [28]:

(2) $$D={\mathrm{RT}\delta }^{-1}\eta_{0}e^{\frac{{\mathrm{pV}}_{0}}{\mathrm{RT}}}$$

where δ is the atomic free path length (m); η_0_ is the atmospheric viscosity at the same temperature (Pa·s); V_0_ is the volume of the atmosphere at the same temperature (m^3^/mol).

The formula shows that the solute diffusion coefficient is affected by the atomic free stroke length and pressure. When the pressure is increased, the atomic free stroke length will decrease, and both will reduce the solute diffusion coefficient. It can be concluded that high pressure will inhibit the solute diffusion.

Because high pressure inhibits the diffusion of solute elements, on the one hand, the solid solubility of the alloy is greatly improved, which will aggravate the solute enrichment at the solidification interface and increase the undercooling during solidification, which is helpful to obtain the solidification structure with uniform distribution. On the other hand, the slurry is in a state of solid–liquid mixing in the semi-solid extrusion process. After extrusion, it will produce large plastic deformation, resulting in large lattice distortion, vacancy, and other defects [29]. With the increase in Mg content, the solid solution of solute elements in the matrix is further promoted, and the trend of segregation and aggregation of alloy elements increases. Therefore, the precipitated phase size of the alloy with a content of 2.5 wt.% Mg is larger than that of the alloy with a content of 0.5 wt.% Mg.

### 3.3. Mechanical Performances for Al−17Si−4Cu−xMg Alloys

**Figure 10:**
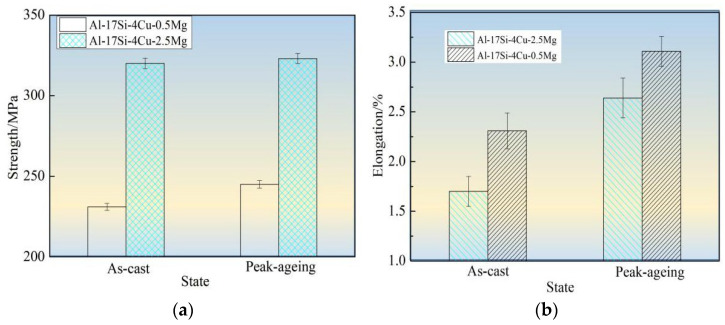

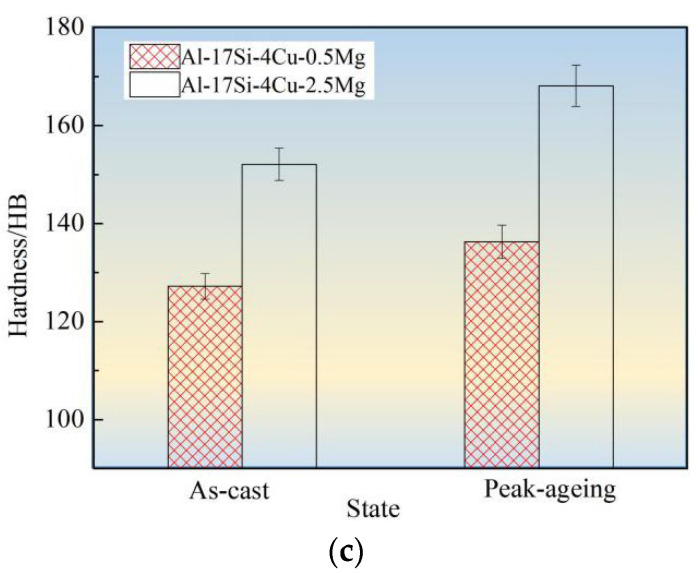
Mechanical performances for hypereutectic Al−17Si−4Cu−xMg alloys. (**a**) Tensile strength; (**b**) Elongation; (**c**) Hardness.

Figure 10 shows tensile strength, elongation, and hardness values for the hypereutectic Al−17Si−4Cu−0.5Mg and Al−17Si−4Cu−2.5Mg alloys in as-cast and peak-ageing states for semisolid extrusion. The final values considerably increased following T6 thermal treatment. Following T6 treatment, those values for Al−17Si−4Cu−0.5Mg reached 331 MPa, 3.11%, and 152.1 HB, respectively. These values exhibited an increase of 35.1%, 17.8%, 19.6%, respectively, compared with those obtained for the unheated state. In addition, the hardness elevated as Mg level within thixoformed Al−Si−Cu−Mg alloys increased. For Al−17Si−4Cu−2.5Mg, its maximum hardness can reach 168.1 HB after the heat treatment, but the elongation and tensile strength decreased as Mg level increased.

Mg solubility in the matrix is 0.1–0.15% and 0.5–0.7% at room and high temperatures, respectively [11]. After adding a small amount of Mg into a hypereutectic Al−Si−Cu alloy, the Q phase formed can enhance the alloy strength. After the heat treatment, a part of Mg can be dissolved in the matrix, leading to lattice distortion. A large portion of the finely dispersed θ′ phase and Q′ metastable phase precipitates. These phases hinder the movement of dislocations and improve alloy strength and hardness. When the Mg content is considerably high, another part of an undissolved phase exists at the grain boundary. This phase leads to a decrease in the bonding strength between grains and becomes the hard–brittle phase. The alloy hardness increases as Mg level increases, mainly because of precipitation of Mg_2_Si and Al_5_Cu_2_Mg_8_Si_6_ phases during hardening at equilibrium and within the solid solution. The precipitated phase number elevates as the Mg level elevates. This phenomenon explains the relation between precipitate content and hardness increase. As the Mg level reaches 2.5 wt.%, the second phase further increases in the alloy structure. The increase in the Mg content promotes Al_5_Cu_2_Mg_8_Si_6_, Al_8_FeMg_3_Si, and Mg_2_Si phase coarsening and enhances the cracking effect on the matrix [29,30]. After the ageing treatment, the size of precipitated acicular β′, θ′, and Q′ phases increases. Dislocation is mainly a bypass mechanism, and the effect of strengthening of Mg on the alloy reduces, thus leading to decreased alloy elongation and tensile strength.

**Figure 11:**
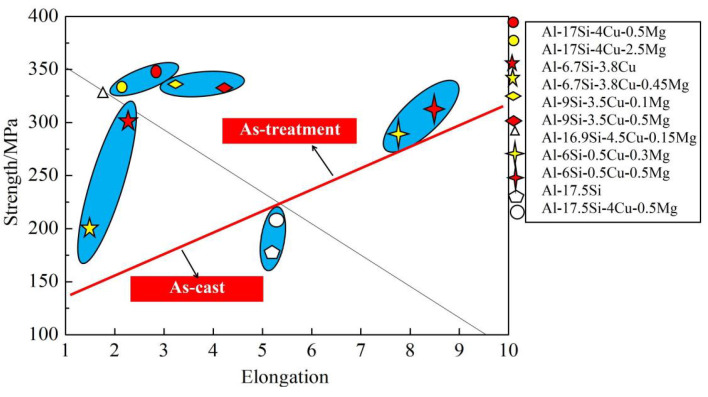
Mechanical performances compared among different Al−Si alloys.

Figure 11 compares mechanical performances between our as-cast and other alloys [8,9,19,31,32]. In a certain range, the increase in Mg content can substantially improve the alloy strength (Figure 11). In this experiment, for the Al−17Si−4Cu−0.5Mg alloy, its elongation and tensile strength reached 3.11% and 331 MPa, respectively, and those of the Al−17Si−4Cu−2.5Mg alloy attained 323 MPa and 2.61%, respectively, after the T6 thermal treatment. Our studied alloys have increased strength compared with most Al−Si alloys, mainly because of the adoption of semisolid extrusion which substantially refines the alloy microstructure.

**Figure 12:**
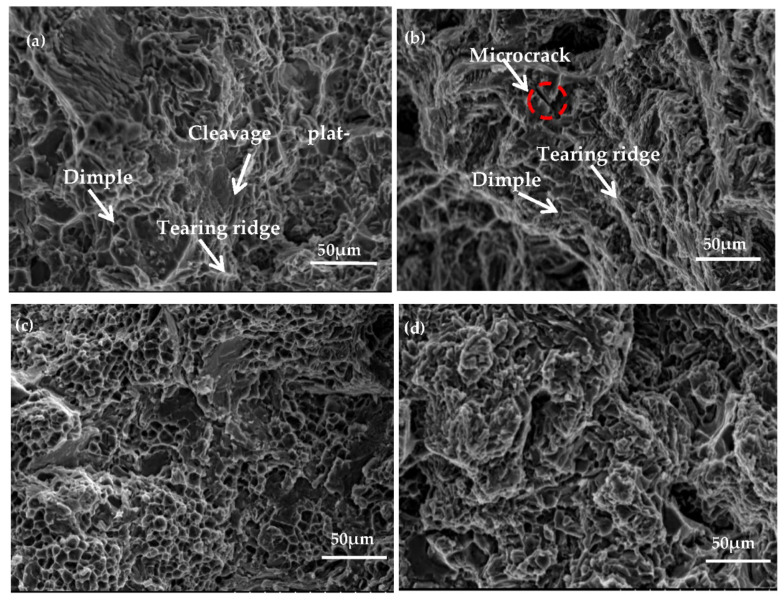
Fracture morphology for the hypereutectic Al−17Si−4Cu−xMg alloy: (**a**,**c**) Al−17Si4Cu−0.5Mg under as-cast state under and peak-ageing state. (**b**,**d**) Al−17Si−4Cu−2.5Mg under as-cast state under and peak-ageing state.

Figure 12a,c presents the tensile fracture morphology of Al−17Si−4Cu−0.5Mg under as-cast and peak ageing conditions, respectively. The alloy fracture mode is perpendicular to the tensile direction. Following T6 thermal treatment, cleavage platform size decreases, and the number of dimples increases; dimples are equiaxed and deep. Figure 12b,d presents the 2.5 wt.% Mg alloy fracture morphology. The cleavage plateau size of the alloy with the high Mg content considerably increases compared with that with low Mg content (0.5 wt.%). Microcracks can be seen on the cleavage platform surface. Because there are great inclusions within the alloy, this material becomes prone to stress concentration when force is applied, causing the matrix around the inclusion phase to produce microcracks, or causing the inclusion phase to rupture. Through the direct connection of microcracks, cracks are rapidly generated between micropores, resulting in a large decrease in elongation, and material toughness is poor.

## 4. Conclusions

This study analyzed how the formation of Mg_2_Si affects the microstructure and mechanical properties for Al−17Si−4Cu−xMg (x = 0.5 and 2.5) alloys. The major conclusions are drawn below:

1. According to an equilibrium phase diagram calculation, when x > 2.2 wt.%, Mg_2_Si phase appears in the hypereutectic Al−17Si−4Cu−xMg alloys formed through semisolid extrusion. The Al−17Si−4Cu−2.5Mg microstructure revealed that the diffraction peak of the Mg_2_Si phase appears in the XRD diffraction curve, and the Mg_2_Si phase of Chinese characters is observed in the SEM, which is consistent with the calculation results. After the T6 heat treatment, short rod-like θ′ and needle-like Q′ phases formed within the Al−17Si−4Cu−0.5Mg alloy. In addition, needle-like and dot-like β″ phases were observed in the Al−17Si−4Cu−2.5Mg alloy.
2. According to the DSC solidification curve and solidification phase diagram, during solidification, the hypereutectic Al−17Si−4Cu−xMg alloy mainly follows the path: L → α-Al + eutectic Si + Q phase + θ phase with Mg content < 2.2 wt.%, and L → α-Al + eutectic Si + Q phase + θ phase + β phase with Mg content > 2.2 wt.%.
3. The mechanical properties were considerably improved following aging and solution treatments on semisolid extrusion Al−17Si−4Cu−xMg alloys. For the peak ageing treatment, values of tensile strength, elongation, as well as hardness for Al−17Si−4Cu−0.5Mg were 331 MPa, 3.11%, and 168.1 HB, respectively. After the Mg level was elevated to 2.5 wt.%, tensile strength and elongation decreased, but hardness increased mainly because the hard–brittle phases of Mg_2_Si, Al_8_FeMg_3_Si, and Al_5_Cu_2_Mg_8_Si_6_ are formed in the alloy.

## Figures and Tables

**Table 1:** Chemical compositions of the studied alloys (wt.%).

Alloy	Si	Cu	Mg	Fe	Al
1	17.49	4.26	0.48	0.19	Bal.
2	17.25	3.97	2.45	0.24	Bal.

**Table 2:** DSC remelting characteristics of the as-cast alloy samples.

Alloys	Temperature of Peak1/°C		Temperature of Peak2/°C		Temperature of Peak3/°C		Temperature of Peak4/°C	
	Onset	Peak	Onset	Peak	Onset	Peak	Onset	Peak
Al−17Si−4Cu−0.5Mg	495.3	521.0	501.5	537.3	544.5	590.3	—	—
Al−17Si−4Cu−2.5Mg	497.6	518.1	522.6	531.7	529.2	555.2	540.9	578.3

**Table 3:** The microstructure of the alloy after solidification corresponding to different Mg content calculated by the Scheil module.

W(Mg)/%	Microstructure after Solidification
0.5	α-Al + eutectic Si + Q-Al_5_Cu_2_Mg_8_Si_6_ + θ-Al_2_Cu
2.5	α-Al + eutectic Si + Q-Al_5_Cu_2_Mg_8_Si_6_ + θ-Al_2_Cu+ β-Mg_2_Si

**Table 4:** EDS analyses on various phases observed from Figure 7 (at %).

Locations	Al	Si	Cu	Mg	Fe	Phase	Morphology
A	75.09	18.85	2.77	3.29	—	Q(Al_5_Cu_2_Mg_8_Si_6_)	Gridded
B	74.99	—	25.01	—	—	θ(Al_2_Cu)	Globular
C	71.26	16.42	—	5.23	6.09	β(Al_8_FeMg_3_Si)	Strip
D	68.15	26.14	—	5.71	—	β(Mg_2_Si)	Chinese characters

## Data Availability

The data used to support the findings of this study are available from the corresponding author upon request.
